# Simple and efficient machine learning frameworks for identifying protein-protein interaction relevant articles and experimental methods used to study the interactions

**DOI:** 10.1186/1471-2105-12-S8-S10

**Published:** 2011-10-03

**Authors:** Shashank Agarwal, Feifan Liu, Hong Yu

**Affiliations:** 1 Medical Informatics, College of Engineering and Applied Sciences, University of Wisconsin-Milwaukee, Milwaukee, WI, USA; 2 Department of Health Sciences, College of Health Science, University of Wisconsin-Milwaukee, Milwaukee, WI, USA; 3 Department of Computer Science and Electrical Engineering, College of Engineering and Applied Sciences, University of Wisconsin-Milwaukee, Milwaukee, WI, USA

## Abstract

**Background:**

Protein-protein interaction (PPI) is an important biomedical phenomenon. Automatically detecting PPI-relevant articles and identifying methods that are used to study PPI are important text mining tasks. In this study, we have explored domain independent features to develop two open source machine learning frameworks. One performs binary classification to determine whether the given article is PPI relevant or not, named “Simple Classifier”, and the other one maps the PPI relevant articles with corresponding interaction method nodes in a standardized PSI-MI (Proteomics Standards Initiative-Molecular Interactions) ontology, named “OntoNorm”.

**Results:**

We evaluated our system in the context of BioCreative challenge competition using the standardized data set. Our systems are amongst the top systems reported by the organizers, attaining 60.8% F1-score for identifying relevant documents, and 52.3% F1-score for mapping articles to interaction method ontology.

**Conclusion:**

Our results show that domain-independent machine learning frameworks can perform competitively well at the tasks of detecting PPI relevant articles and identifying the methods that were used to study the interaction in such articles.

**Availability:**

Simple Classifier is available at http://sourceforge.net/p/simpleclassify/home/ and OntoNorm at http://sourceforge.net/p/ontonorm/home/.

## Introduction

Protein-protein interactions (PPI) are responsible for many biological phenomena. Understanding these interactions can greatly benefit biological research; for example, it can help us understand causes of certain diseases which can in turn lead to development of therapeutic interventions. A case of significance of protein-protein interactions can be seen for the BRCA1 and BARD1 proteins, which have been reported to interact with each other and a mutation in BRCA1 can disrupt this interaction, which can lead to breast cancer [1].

The importance of PPIs has led to the development of several curated databases including IntAct [2], BioGRID [3] and MINT [4]. These databases are generally curated manually by humans and store information including the proteins that interact with each other, the articles in which these interactions were detected and the methods that were used to discover these interactions. However, manually curating articles for PPIs is a time consuming process and due to the fast rate of research and rapid increase in amount of published literature, the amount of effort required to maintain such databases has increased significantly. This has spurred the development of text-mining approaches to automate identification of such interactions and help the manual curation process.

One of the important tasks is to identifying the methods used to study PPIs, known as the interaction method task (IMT). IMT helps database curators determine the validity of the reported interactions. Certain methods give better evidence of an interaction than others [5,6]. The methods sub-ontology in the PSI-MI (Proteomics Standards Initiative-Molecular Interactions) ontology is a controlled vocabulary to which interaction methods can be mapped [7]. Annotating methods with PSI-MI’s methods sub-ontology will help database curation efforts.

To efficiently identify PPI interaction methods, another important task is to first determine if the given article contains protein-protein interaction or not, known as article classification task (ACT). ACT is indispensable for other PPI related text mining applications, such as interaction event detection.

Different approaches have been developed for the ACT task. A simple approach is to make use of n-gram features to train supervised machine learning algorithms, which have been deployed in many similar tasks [8-19]. Normalization and feature selection may be conducted before training the classifiers. Domain-specific adaptations of this approach have been used for this task as well. An modification was proposed to make use of contextual bag-of-words [20]. The context information included the number of protein names that appear in the abstract of an article to be classified with the assumption that the presence of more protein names in the abstract indicates greater likelihood that the article contains protein-protein interaction data. Support vector machine (SVM) classifiers were trained on these contextual bag-of-words features. Other extension work added MeSH terms as features along with selected n-gram features [21-24]. Grover et al. used a “bag-of-nlp” approach where the output of a natural language processing pipeline was augmented with word features to classify articles [25]. Dogan et al. identified the 10-nearest neighbors in the training data of the test article and used the gold standard annotation of these 10-nearest neighbors as features along with the n-gram features [26].

Approaches that explore features beyond words for classification training have also been proposed. A semi-supervised approach was suggested by [27], where dependency tree based patterns are automatically learned from the training data. A set of eight patterns were manually seeded for this approach. Another approach made use of information retrieval techniques to identify protein-protein extraction relevant documents [28]. A set of well-known protein interaction related keywords is used as queries. An approach by Kolchinsky et al. made use of features from citation network of relevant literature to classify articles [15]. Kim and Wilbur extracted automatically grammatical patterns from the training corpus and used these patterns for ACT [29]. They found that this approach performs better than the machine learning approaches that were based on bag-of-words representation.

Although a lot of research has been done for the ACT, research for identifying interaction methods is limited. Similar to our goal, most studies in this area attempt to associate method nodes in the PSI-MI ontology to articles. The OntoGene system developed by Rinaldi et al. [23,30] makes use of pattern matching techniques to identify interaction methods. The system makes use of handcrafted patterns to improve performance. Pattern matching approach has be employed by Lourenco et al. [31] as well. Dogan et al. combined pattern matching and k-nearest neighbors’ annotations for this task [26]. They also mapped the article’s MeSH terms to PSI-MI nodes to identify relevant method nodes. Use of machine learning-based approaches that view IMT as a document-level classification problem have been reported [24,32]. They expanded the synonyms for PSI-MI nodes by adding synonyms from UMLS Metathesaurus. Matos et al. approached IMT as an information retrieval problem [19]. The documents were indexed using Lucene and retrieved using method names.

In this study, we report on the development of machine learning frameworks to identify articles that contain protein-protein interaction data and then process these articles to identify the methods that were used to discover protein-protein interactions. Unlike previous approaches many of which rely on human curated data or domain-specific features, our goal is to develop an adaptable framework by exploring domain independent features, which can be generalized to other text mining applications with no or minimum adaptation. For example, our ACT framework can be applied to train and classify any type of text documents, regardless of the domain they belong to. Similarly, our IMT framework can be used to map terms from any ontology to any text. As a result, we explored machine learning-based approaches using features that are domain independent.

The BioCreative (Critical Assessment of Information Extraction systems in Biology) challenge is a community effort to promote the development of biomedical text mining applications. Till date, four BioCreative challenges have been organized. Interaction methods featured in two of these challenges while article classification task featured in the last three challenges [33-37]. The latest BioCreative challenge, BioCreative III, includes both ACT and IMT. We used the data and evaluation provided through BioCreative III to develop and evaluate our machine learning frameworks.

## Methods

We explored supervised machine learning approaches for both ACT and IMT. The data we used for training is described below, followed by the ACT classification and IMT classification tasks.

### Training, development and test data

The organizers of BioCreative III provided training data, development data and test data for both tasks [38]. The size of the data provided for ACT and IMT is mentioned in Table 1 and Table 2, respectively. Note that the data provided for ACT and IMT are independent of each other. For ACT, the distribution of positive and negative instances in the development data reflected the true distribution of positive and negative instances, i.e. approximately 16% of the articles contained protein-protein interactions data. For ACT training data, equal number of positive and negative instances were provided. The distribution of instances in the test data was similar to the distribution of instances in the development data. The article’s title and abstract were used for training and testing.

**Table 1:** ACT Data

	ACT Data (article abstracts)
Training Data Total	2280
Training Data Positive	1140
Training Data Negative	1140
Development Data Total	4000
Development Data Positive	682
Development Data Negative	3318
Test Data Total	6000

The organizers of BioCreative III provided training, development and test data for ACT. The size of the data is shown in this table.

**Table 2:** IMT Data

	IMT Data (article full-texts)
Training Number of Articles	2035
Training Number of Annotations	4347
Training Annotations per Article	2.14
Development Number of Articles	587
Development Number of Annotations	1379
Development Annotations per Article	2.35
Test Number of Articles	305

The organizers of BioCreative III provided training, development and test data for IMT. The size of the data is shown in this table.

For IMT, the task was to identify interaction methods at a document level and not at interaction or mention level. The methods sub-ontology of the PSI-MI ontology was used to obtain the collection of possible methods. From this sub-ontology, 115 nodes were allowed for IMT. Four nodes from the 115 allowed nodes accounted for roughly half of all annotations; these were (in order of highest to lowest frequency): “anti-bait coimmunoprecipitation”, “anti-tag coimmunoprecipitation”, “pull down” and “two hybrid”. “Anti-bait coimmunoprecipitation” and “anti-tag coimmunoprecipitation” accounted for one third of all annotations. Within the test data, 222 articles out of the 305 articles were annotation-relevant; hence the remaining 83 articles had no annotations assigned to them. The full-text of the article was used for training and testing. Although the full-text articles were originally in PDF format, the organizers of BioCreative III also provided the corresponding files in text format, which were used for training and testing in our experiments.

### ACT Classification

As noted earlier, the distribution of data in the development data is similar to the distribution of test data. Hence, for tuning, we trained models on the development data and tested them on the training data. We trained two different classifier models – Support Vector Machines (SVM) with polynomial kernel [39] and Naïve Bayes Multinomial (NBM). We used the implementation provided in the Weka data mining library [40] (downloaded from: http://www.cs.waikato.ac.nz/ml/weka/).

We normalized all text by lowercasing all characters, removing punctuations, stemming all words (using Porter stemming algorithm [41,42]) and removing numbers. We then extracted unigrams (individual words) and bigrams (two consecutive words) as features. As this led to a large number of features, we conducted feature selection with two feature scoring algorithms: mutual information and chi-square score. All features were scored with these algorithms and we used the top 20, 50, 100, 400 and 1000 features to train the classifier.

We explored various combinations of different classifier algorithms, feature selection methods and feature numbers mentioned above. We tried using unigrams only as well as using both unigrams and bigrams. All features were uniformly weighted when provided to the machine learning classifiers. For the BioCreative III challenge, we were allowed to submit 10 runs for ACT. The runs are listed in Table 3. For six of the ten runs, we combined the training and development data to train the classifier, as we expected larger training data to perform better. At the same time, the distribution of the development data was similar to that in the test data; hence, for the remaining four runs, we trained the classifier on development data only.

**Table 3:** Runs submitted for ACT

Run number	Label	Classifier Algorithm	Type of features used	Number of features	Training data
1	NBM-12-1k-td	NBM	Unigrams and Bigrams	1000	Training+Development
2	NBM-12-400-td	NBM	Unigrams and Bigrams	400	Training+Development
3	NBM-12-1k-d	NBM	Unigrams and Bigrams	1000	Development
4	SVM-12-400-d	SVM	Unigrams and Bigrams	400	Development
5	SVM-12-400-td	SVM	Unigrams and Bigrams	400	Training+Development
6	NBM-1-1k-td	NBM	Unigrams	1000	Training+Development
7	NBM-1-400-td	NBM	Unigrams	400	Training+Development
8	NBM-1-1k-d	NBM	Unigrams	1000	Development
9	SVM-1-400-d	SVM	Unigrams	400	Development
10	SVM-1-400-td	SVM	Unigrams	400	Training+Development

For the BioCreative III challenge, each participating team was allowed to submit 10 runs for ACT. Five runs could be submitted offline and the other five runs could be submitted online, using XML-RPC. Runs 1-5 were submitted offline, while runs 6-10 were submitted online. For all runs, we used mutual information feature selection algorithm, as it gave better performance than chi-square score. We submitted 10 runs, listed here.

For ACT, we developed a framework that can apply the feature selection methods described above with different classifier algorithms. The framework is called SimpleClassifier and is available online at http://sourceforge.net/p/simpleclassify/home/. It can be used to train classifiers for any text collection.

### IMT Classification

The IMT involved mapping nodes in PSI-MI ontology to articles. For each ontology node, we obtained the concept name and its synonyms. We manually added synonyms for some ontology nodes, such as “anti bait immunoprecipitation” for “anti bait coimmunoprecipitation” and “radioligand binding” for “saturation binding”. A keyword for each ontology node was manually extracted by the first author, for example, “coimmunoprecipitation” for “anti bait coimmunoprecipitation”. The keywords were selected based on the author’s judgment of the most informative word in the concept. We extracted unigrams and bigrams from each node’s concept name and synonyms. For each unigram and bigram, we calculated the mutual information score and chi-square value using the training data. The top 10 unigrams and bigrams by mutual information score and chi-square value are displayed in Table 4.

**Table 4:** Top 10 Unigrams and Bigrams for IMT

Term	Mutual information score	Chi-square value
two hybrid	0.439	1225.574
immunoprecipitation	0.437	1110.124103
hybrid	0.398	1041.587496
yeast two	0.348	1061.789
diffraction	0.263	1142.337
resonance	0.236	969.286
crystallography	0.182	751.011
x ray	0.176	622.764
yeast	0.173	402.283
gal4	0.168	576.122

The top 10 unigrams and bigrams by mutual information score and their corresponding chi-square values. The terms are sorted by their mutual information score.

We approached IMT as a classification task, where we try to determine if an article-ontology node pair is positive or negative. We identified 21 features (as listed in Table 5) and scored those features for each article-ontology node pair. We then trained machine learning classifiers Random Forest [43], Random Committee [44], Naive Bayes Tree [45] and J48 [46] to predict the label for each article-ontology node pair. All features were uniformly weighted when provided to the machine learning classifiers.

**Table 5:** Features used for IMT

Feature	Feature type	Description
Perfect match (2 features)	Binary	For each node, checks if (1) the concept name or (2) any synonym name appears in the article
Term match (4 features)	Binary	For each node, checks if any unigram/bigram in the node’s (1, 2) concept name or (3, 4) synonyms appears in the article
Term match ratio (4 features)	Continuous	For each node, the ratio unigram/bigram in the node’s (1, 2) concept name or (3, 4) synonyms that appears in the article
Matched terms mutual information sum (4 features)	Continuous	Sum of mutual information score of each matching uni-gram/bigram in the node’s (1, 2) concept name or (3, 4) any synonym.
Matched term chi-squared sum (4 features)	Continuous	Sum of chi-squared value of each matching unigram/bigram in the node’s (1, 2) concept name or (3, 4) any synonym.
Node popularity	Integer	The number of times this node is annotated in the training data
Regex annotation	Binary	Checks if the regular expression-based annotator that was provided by the organizers of BioCreative III annotates the current article-ontology node pair
Keyword presence	Binary	Checks if the keyword for the ontology node appears in the article

We then conducted feature selection using the chi-square measure which comes with Weka’s built-in feature selection module. To identify the best feature set, we tuned the classifiers by training on training data and testing on development data. We counted the number of true positives, false positives, false negatives and true negatives. An article-node pair was considered to be true positive if both gold-standard and the classifier identified the pair to be positive. A pair was considered to be false positive if the gold standard did not consider the pair to be positive, but the classifier did. A pair was considered to be false negative if the gold standard considered the pair to be positive, but the classifier did not. A true negative was marked if both the gold standard and the classifier considered the pair to be negative. Using these counts, we calculated the precision and recall with the following formulae -

From the recall and precision, the F1-Score was calculated by taking their harmonic mean. The F1-Score obtained was used as a measure of performance during the parameter tuning process, by which we obtained the best number of features for each classifier.

For each article, we identified the evidence sentence from which each interaction method was identified. For this, we calculated a score for each sentence, and the sentence with the highest score was considered to be associated with the interaction method. To calculate the score, the unigrams in the interaction method’s name were looked for in each sentence. If a unigram was present in the sentence, then the unigram’s chi-square value was added to the sentence’s score. If no unigrams were present in the sentence, then a score of 0 was assigned to the sentence. If multiple sentences had the same score, the longest sentence was associated with the interaction method.

As a result, we developed a framework for IMT that can make use of the features mentioned above and conduct feature selection. The framework is called OntoNorm and is available online at http://sourceforge.net/p/ontonorm/home/. It can be used to train models with any ontology and text collection.

Similar to ACT, we were allowed to submit 10 runs for IMT. The runs are listed in Table 6. We trained the classifier on the combination of both training and development data for the runs.

**Table 6:** Runs submitted for IMT

Run number	Label	Algorithm	Number of features
1	j48-21	J48	All (21 features)
2	rc-21	Random Committee	All (21 features)
3	rf-21	Random Forest	All (21 features)
4	j48-14	J48	14 features
5	rf-12	Random Forest	12 features
6	rc-12	Random Committee	12 features
7	rc-14	Random Committee	14 features
8	rf-7	Random Forest	7 features
9	nbt-7	Naïve Bayes Tree	7 features
10	rf-15	Random Forest	15 features

For the BioCreative III challenge, each participating team was allowed to submit 10 runs for IMT. Five runs could be submitted offline and the other five runs could be submitted online, using XML-RPC. Runs 1-5 were submitted offline, while runs 6-10 were submitted online. For all runs, we combined the training and the development data. We submitted 10 runs, listed here.

### Evaluation

As mentioned earlier, we participated in the BioCreative III challenge for evaluation. The evaluation of the runs was conducted by the organizers. Micro-averaged F1-score, Matthew’s Correlation Coefficient and AUC iP/R were used as evaluation metrics. Matthew’s Correlation Coefficient is a measure of binary classification and is based on the chi-squared statistic obtained for a 2x2 contingency table. It is measured by the following formula -

where n is the total number of observations. The area under the curve (AUC iP/R) was measured by drawing the precision/recall curve and interpolating the curve. The area under this curve is the AUC iP/R.

For ACT, the accuracy, sensitivity and specificity of the system were also measured. The accuracy is the ratio of correctly classified instances and all instances, sensitivity is the ratio of true positive instances and all positive instances and specificity is the ratio of true negative instances and all negative instances.

## Results

When tuning, we observed that for ACT, the best F1-Scores were obtained when using 400 or 1000 features with Naïve Bayes Multinomial (NBM) and 400 features with Support Vector Machines (SVM) (Additional File 1). The top 10 features (unigrams and bigrams) were: ‘interact’, ‘interact with’, ‘bind’, ‘protein’, ‘domain’, ‘bind to’, ‘phosphoryl’, ‘regul’, ‘complex’ and ‘activ’. Similarly, for IMT the best results were seen when using 14 features with J48, 7 features with Naïve Bayes Tree, 12 features with Random Forest and 12 features with RandomCommittee (Additional File 2). The top five features were: ‘unigram chi-square sum’, ‘unigram mutual information sum’, ‘unigram chi-square sum concept only’, ‘unigram mutual information sum concept only’ and ‘bigram mutual information sum’. Our runs try to cover all algorithms and different feature combinations. For ACT and IMT, the performance of our runs is shown in Table 7 and Table 8, respectively. For ACT, we found that performance of SVM-based classifiers was better than NBM-based classifiers, although during tuning we found that NBM-based classifiers performed better. For IMT, the result of all classifiers were very close to each other; F1-score difference between best and worst runs was less than 2.5% points. We found that runs for which feature selection was done performed better than the runs for which all features were used, indicating that certain features are not useful.

**Table 7:** ACT Results

Run number	Label	Accuracy (%)	Specificity (%)	Sensitivity (%)	F1-Score (%)	MCC	AUC iP/R (%)
1	NBM-12-1k-td	80.02	80.90	75.06	53.26	0.449	61.29
2	NBM-12-400-td	81.00	81.75	76.81	55.08	0.472	62.13
3	NBM-12-1k-d	82.40	83.85	74.29	56.15	0.482	60.48
4	SVM-12-400-d	87.73	94.79	48.24	54.40	0.480	43.76
5	SVM-12-400-td	87.27	91.81	61.87	59.58	0.521	48.47
6	NBM-1-1k-td	77.80	77.84	77.58	51.46	0.432	57.44
7	NBM-1-400-td	78.05	78.15	77.47	51.71	0.434	57.56
8	NBM-1-1k-d	79.90	81.00	73.74	52.67	0.441	54.97
9	SVM-1-400-d	86.25	92.06	53.74	54.24	0.462	41.58
10	SVM-1-400-td	86.87	90.39	67.14	60.80	0.533	47.40

The result of submitted ACT runs on the test data. Legend: MCC=Matthew’s correlation coefficient

**Table 8:** IMT Results

Run	Label	Precision (%)	Recall (%)	F1-Score (%)	MCC	AUC iP/R (%)
1	j48-21	52.52	49.53	50.98	0.500	28.20
2	rc-21	52.02	48.96	50.44	0.495	28.59
3	rf-21	50.78	49.34	50.05	0.490	27.24
4	j48-14	52.50	49.91	51.17	0.502	29.22
5	rf-12	52.58	52.18	52.38	0.514	29.98
6	rc-12	52.71	51.61	52.16	0.512	29.93
7	rc-14	52.28	50.10	51.16	0.502	30.05
8	rf-7	52.28	52.18	52.23	0.512	30.05
9	nbt-7	49.55	52.56	51.01	0.500	29.30
10	rf-15	51.76	50.29	51.01	0.500	29.80

The result of submitted IMT runs on the test data. Legend: MCC=Matthew’s correlation coefficient

### Results in relation to other systems

We compared the performance of our system with other teams that participated in ACT and IMT tasks for the BioCreative III challenge. Ten teams participated in ACT and eight in IMT. For ACT, compared to other participants, our system’s ranked 2nd when measured by F1-Score and Matthew’s Correlation Coefficient vale, and 5th when measured by AUC iP/R and accuracy. The best performing systems from any team for these measures attained 89.15% accuracy, 61.42% F1-Score, 0.553 Matthew’s Correlation Coefficient value and 67.98% AUC iP/R. Our best performance on these measures was: 87.73% accuracy, 60.80% F1-Score, 0.533 Matthew’s correlation coefficient value and 62.13% AUC iP/R. These results indicate that the performance of our systems was very close to the performance of the best performing system.

Similarly for IMT, our system ranked 3rd when measured by F1-Score, Matthew’s Correlation Coefficient value and AUC iP/R. The best performing systems from any team for these measures attained 55.12% F1-Score, 0.542 Matthew’s Correlation Coefficient value and 35.42% AUC iP/R. Our best performance on these measures was: 52.38% F1-Score, 0.514 Matthew’s Correlation Coefficient value and 30.05 % AUC iP/R.

## Discussion

We have developed supervised machine learning frameworks to identify articles that contain protein-protein interaction data and to map ontology nodes to text of an article. Our goal was to develop these approaches independent of domain knowledge and manual intervention, such that they can be viewed as frameworks that can be applied to other article classification task and ontology mapping tasks. For ACT, our system, Simple Classifier, meets these goals. For IMT, we did modify the ontology by manually adding synonyms and keywords, because of which we cannot claim that OntoNorm meets our goal of being free from manual intervention; however, given an ontology with comprehensive list of synonyms, this manual intervention would be unnecessary. In this sense, OntoNorm can be used to map terms from any given ontologies to any text articles.

For ACT, our approach was simpler than the approach used by many other teams at the BioCreative challenge. Despite the simplicity, our system ranked 2^nd^ amongst 10 teams, and the difference between the performance of the team that ranked 1^st^ and our system was marginal, suggesting that the frameworks we employed in this study are very efficient, competitive and robust. Our SVM-based runs obtained poor AUC iP/R, despite of obtaining good accuracy and F1-score. This was because for most instances, the annotation confidence assigned by the classifier was 100%, which prevented the results to be ranked meaningfully. Except for AUC iP/R, SVM-based models performed better than NBM-based models on the test data, although NBM-based models performed better during tuning. This maybe because the NBM-based models overfit on the training data.

On analyzing incorrectly classified ACT cases, we observed that false positives were seen when an article contained terms that usually indicate protein-protein interaction, but were not used in that context; for example, the article with PMID:19694809 uses the keyword ‘interaction’, but does not indicate protein-protein interaction in this context. At the same time, false negatives were seen when such terms were missing, although the article contained protein-protein interaction data; for example, article with PMID:19724778. The error analysis uncovers one disadvantage of our machine-learning framework that it is based only on lexical features, which may not contain sufficient information and can cause ambiguities in some cases. It also suggests that deep linguistic analysis (e.g. syntactic and semantic analysis) might be needed to further enhance the system’s performance.

For IMT, we identified several domain independent features to classify article-node pairs. We believe that the approach works well, as our system was placed 3^rd^ amongst 8 teams at BioCreative III. We found that tree-based classifier algorithms such as Random Forest and J48 performed better at this task. Most of our errors were seen when annotating nodes “anti-tag coimmunoprecipitation” and “anti-bait coimmunoprecipitation” as “coimmunoprecipitation” was usually mentioned in relevant articles, but whether it was anti-tag or anti-bait coimmunoprecipitation was not explicitly stated. For example, article [47] was falsely annotated with anti tag coimmunoprecipitation.

We found that unigram related features ranked higher than bigram related features in the IMT task, as 4 out of 5 top features are from unigrams. We speculate that this is because of the high variance when discussing different interactive methods in articles, such that unigram features become more reliable than bigrams.

## Conclusion

We have developed machine learning frameworks that make use of domain independent features to classify text (Simple Classifier) and to map nodes in an ontology to text (OntoNorm). These frameworks obtain competitive performance compared with other participant teams when applied on tasks to identify articles that contain protein-protein interaction data and to identify methods from an ontology that were used to study these interactions.

In the future, we may apply our frameworks on other text mining applications. In addition, our current approach for OntoNorm does not make use of the hierarchy of the ontology, which will be investigated and evaluated in the future as well.

## Authors' contributions

SA conducted the experiments. HY and FL provided guidance.

## Competing interests

The authors declare that they have no competing interests.

## Supplementary Material

Additional file 1 **ACT Tuning data** Results of various classifier algorithms, feature selection algorithms and number of features combinations when trained on ACT development data and tested on ACT training data Click here for file

Additional file 2 **IMT Tuning data** Results of various classifier algorithms, feature selection algorithms and number of features combinations when trained on ACT development data and tested on ACT training data Click here for file
