# Supplementary material for: Simple and efficient machine learning frameworks for identifying protein-protein interaction relevant articles and experimental methods used to study the interactions
Source: BMC Bioinformatics. 2011 Oct 3;12(Suppl 8):S10. doi: 10.1186/1471-2105-12-S8-S10 (PMC3269933; doi:10.1186/1471-2105-12-S8-S10)
Supplement: Additional file 1 — ACT Tuning data Results of various classifier algorithms, feature selection algorithms and number of features combinations when trained on ACT development data and tested on ACT training data [file 1471-2105-12-S8-S10-S1.docx]

ACT Tuning data: Results of various classifier algorithms, feature selection algorithms and number of features combinations when trained on ACT development data and tested on ACT training data

| Classifier | Types of features | Feature selection algorithm | Number of features | Recall | Precision | F1-score |
| --- | --- | --- | --- | --- | --- | --- |
| NBM | Unigrams and Bigrams | mutual information | 20 | 0.313158 | 0.959677 | 0.472222 |
| NBM | Unigrams and Bigrams | mutual information | 50 | 0.581579 | 0.909465 | 0.70947 |
| NBM | Unigrams and Bigrams | mutual information | 100 | 0.7 | 0.878855 | 0.779297 |
| NBM | Unigrams and Bigrams | mutual information | 400 | 0.842982 | 0.824893 | 0.833839 |
| NBM | Unigrams and Bigrams | mutual information | 1000 | 0.825439 | 0.826163 | 0.825801 |
| NBM | Unigrams and Bigrams | chi squared | 20 | 0.216667 | 0.972441 | 0.354376 |
| NBM | Unigrams and Bigrams | chi squared | 50 | 0.507895 | 0.933871 | 0.657955 |
| NBM | Unigrams and Bigrams | chi squared | 100 | 0.580702 | 0.893387 | 0.703881 |
| NBM | Unigrams and Bigrams | chi squared | 400 | 0.801754 | 0.845513 | 0.823053 |
| NBM | Unigrams and Bigrams | chi squared | 1000 | 0.823684 | 0.831709 | 0.827677 |
| SVM | Unigrams and Bigrams | mutual information | 20 | 0.5 | 0.940594 | 0.652921 |
| SVM | Unigrams and Bigrams | mutual information | 50 | 0.542105 | 0.933535 | 0.685905 |
| SVM | Unigrams and Bigrams | mutual information | 100 | 0.565789 | 0.921429 | 0.701087 |
| SVM | Unigrams and Bigrams | mutual information | 400 | 0.576316 | 0.917598 | 0.707974 |
| SVM | Unigrams and Bigrams | mutual information | 1000 | 0.535965 | 0.882948 | 0.667031 |
| SVM | Unigrams and Bigrams | chi squared | 20 | 0.497368 | 0.935644 | 0.649485 |
| SVM | Unigrams and Bigrams | chi squared | 50 | 0.536842 | 0.927273 | 0.68 |
| SVM | Unigrams and Bigrams | chi squared | 100 | 0.535965 | 0.920181 | 0.677384 |
| SVM | Unigrams and Bigrams | chi squared | 400 | 0.547368 | 0.921713 | 0.686846 |
| SVM | Unigrams and Bigrams | chi squared | 1000 | 0.515789 | 0.88024 | 0.650442 |
| NBM | Unigrams | mutual information | 20 | 0.264912 | 0.906907 | 0.410048 |
| NBM | Unigrams | mutual information | 50 | 0.585088 | 0.903794 | 0.71033 |
| NBM | Unigrams | mutual information | 100 | 0.661404 | 0.891253 | 0.759315 |
| NBM | Unigrams | mutual information | 400 | 0.797368 | 0.847948 | 0.821881 |
| NBM | Unigrams | mutual information | 1000 | 0.841228 | 0.847922 | 0.844562 |
| NBM | Unigrams | chi squared | 20 | 0.20614 | 0.94 | 0.338129 |
| NBM | Unigrams | chi squared | 50 | 0.477193 | 0.918919 | 0.628176 |
| NBM | Unigrams | chi squared | 100 | 0.589474 | 0.904441 | 0.713755 |
| NBM | Unigrams | chi squared | 400 | 0.751754 | 0.87449 | 0.808491 |
| NBM | Unigrams | chi squared | 1000 | 0.774561 | 0.862305 | 0.816081 |
| SVM | Unigrams | mutual information | 20 | 0.414035 | 0.921875 | 0.571429 |
| SVM | Unigrams | mutual information | 50 | 0.517544 | 0.929134 | 0.664789 |
| SVM | Unigrams | mutual information | 100 | 0.539474 | 0.923423 | 0.681063 |
| SVM | Unigrams | mutual information | 400 | 0.558772 | 0.911302 | 0.692768 |
| SVM | Unigrams | mutual information | 1000 | 0.533333 | 0.876081 | 0.663032 |
| SVM | Unigrams | chi squared | 20 | 0.442105 | 0.926471 | 0.598575 |
| SVM | Unigrams | chi squared | 50 | 0.494737 | 0.920065 | 0.643468 |
| SVM | Unigrams | chi squared | 100 | 0.526316 | 0.924499 | 0.670766 |
| SVM | Unigrams | chi squared | 400 | 0.55614 | 0.903134 | 0.688382 |
| SVM | Unigrams | chi squared | 1000 | 0.47807 | 0.88474 | 0.620729 |
