# Supplementary material for: Simple and efficient machine learning frameworks for identifying protein-protein interaction relevant articles and experimental methods used to study the interactions
Source: BMC Bioinformatics. 2011 Oct 3;12(Suppl 8):S10. doi: 10.1186/1471-2105-12-S8-S10 (PMC3269933; doi:10.1186/1471-2105-12-S8-S10)
Supplement: Additional file 2 — IMT Tuning data Results of various classifier algorithms, feature selection algorithms and number of features combinations when trained on ACT development data and tested on ACT training data [file 1471-2105-12-S8-S10-S2.docx]

IMT Tuning data: Results of various classifier algorithms and number of features combinations when trained on IMT training data and tested on IMT development data

| Classifier | Number of features | Precision | Recall | F1-Score |
| --- | --- | --- | --- | --- |
| J48 | 1 | 0.68252 | 0.46306 | 0.55177 |
| J48 | 2 | 0.68266 | 0.46033 | 0.54988 |
| J48 | 3 | 0.67994 | 0.4601 | 0.54883 |
| J48 | 4 | 0.67884 | 0.46122 | 0.54926 |
| J48 | 5 | 0.65472 | 0.59367 | 0.6227 |
| J48 | 6 | 0.65677 | 0.60461 | 0.62961 |
| J48 | 7 | 0.64309 | 0.63569 | 0.63937 |
| J48 | 8 | 0.64403 | 0.64667 | 0.64535 |
| J48 | 9 | 0.64403 | 0.64667 | 0.64535 |
| J48 | 10 | 0.64403 | 0.64667 | 0.64535 |
| J48 | 11 | 0.64373 | 0.64691 | 0.64532 |
| J48 | 12 | 0.68113 | 0.64459 | 0.66236 |
| J48 | 13 | 0.68113 | 0.64459 | 0.66236 |
| J48 | 14 | 0.69434 | 0.64492 | 0.66872 |
| J48 | 15 | 0.69245 | 0.64382 | 0.66725 |
| J48 | 16 | 0.67487 | 0.64268 | 0.65838 |
| J48 | 17 | 0.67487 | 0.64268 | 0.65838 |
| J48 | 18 | 0.67546 | 0.64322 | 0.65894 |
| J48 | 19 | 0.67546 | 0.64322 | 0.65894 |
| J48 | 20 | 0.67546 | 0.64322 | 0.65894 |
| J48 | 21 | 0.67546 | 0.64322 | 0.65894 |
| NBTree | 1 | 0.68627 | 0.54091 | 0.60498 |
| NBTree | 2 | 0.66598 | 0.56146 | 0.60927 |
| NBTree | 3 | 0.65014 | 0.60773 | 0.62822 |
| NBTree | 4 | 0.61785 | 0.61167 | 0.61474 |
| NBTree | 5 | 0.61982 | 0.65878 | 0.6387 |
| NBTree | 6 | 0.5846 | 0.64607 | 0.6138 |
| NBTree | 7 | 0.62538 | 0.67702 | 0.65018 |
| NBTree | 8 | 0.61681 | 0.64988 | 0.63291 |
| NBTree | 9 | 0.62567 | 0.66694 | 0.64564 |
| NBTree | 10 | 0.62443 | 0.66612 | 0.6446 |
| NBTree | 11 | 0.61275 | 0.62827 | 0.62041 |
| NBTree | 12 | 0.62327 | 0.65666 | 0.63953 |
| NBTree | 13 | 0.60895 | 0.66135 | 0.63407 |
| NBTree | 14 | 0.59337 | 0.60164 | 0.59748 |
| NBTree | 15 | 0.43523 | 0.61438 | 0.50952 |
| NBTree | 16 | 0.56942 | 0.59398 | 0.58144 |
| NBTree | 17 | 0.44483 | 0.67452 | 0.53611 |
| NBTree | 18 | 0.42552 | 0.61167 | 0.50189 |
| NBTree | 19 | 0.4231 | 0.60169 | 0.49683 |
| NBTree | 20 | 0.4338 | 0.60138 | 0.50402 |
| NBTree | 21 | 0.43285 | 0.59908 | 0.50258 |
| RandomForest | 1 | 0.66426 | 0.57462 | 0.6162 |
| RandomForest | 2 | 0.66426 | 0.57462 | 0.6162 |
| RandomForest | 3 | 0.6619 | 0.57345 | 0.61451 |
| RandomForest | 4 | 0.66258 | 0.57345 | 0.6148 |
| RandomForest | 5 | 0.66142 | 0.60851 | 0.63387 |
| RandomForest | 6 | 0.66236 | 0.61106 | 0.63568 |
| RandomForest | 7 | 0.6622 | 0.64648 | 0.65424 |
| RandomForest | 8 | 0.66134 | 0.64455 | 0.65284 |
| RandomForest | 9 | 0.66079 | 0.64455 | 0.65257 |
| RandomForest | 10 | 0.6608 | 0.64566 | 0.65315 |
| RandomForest | 11 | 0.66079 | 0.64455 | 0.65257 |
| RandomForest | 12 | 0.67388 | 0.6438 | 0.6585 |
| RandomForest | 13 | 0.67017 | 0.63568 | 0.65247 |
| RandomForest | 14 | 0.66958 | 0.63516 | 0.65191 |
| RandomForest | 15 | 0.67132 | 0.63847 | 0.65448 |
| RandomForest | 16 | 0.67045 | 0.63651 | 0.65304 |
| RandomForest | 17 | 0.66987 | 0.63599 | 0.65249 |
| RandomForest | 18 | 0.6693 | 0.63372 | 0.65102 |
| RandomForest | 19 | 0.67103 | 0.63817 | 0.65419 |
| RandomForest | 20 | 0.66988 | 0.63538 | 0.65217 |
| RandomForest | 21 | 0.67132 | 0.63847 | 0.65448 |
| RandomCommittee | 1 | 0.66527 | 0.56887 | 0.61331 |
| RandomCommittee | 2 | 0.66527 | 0.56887 | 0.61331 |
| RandomCommittee | 3 | 0.66219 | 0.56726 | 0.61106 |
| RandomCommittee | 4 | 0.66287 | 0.56726 | 0.61135 |
| RandomCommittee | 5 | 0.66387 | 0.60784 | 0.63462 |
| RandomCommittee | 6 | 0.66326 | 0.60784 | 0.63434 |
| RandomCommittee | 7 | 0.66386 | 0.64484 | 0.65421 |
| RandomCommittee | 8 | 0.66215 | 0.64151 | 0.65167 |
| RandomCommittee | 9 | 0.66159 | 0.64151 | 0.6514 |
| RandomCommittee | 10 | 0.6613 | 0.63964 | 0.65029 |
| RandomCommittee | 11 | 0.66186 | 0.64069 | 0.6511 |
| RandomCommittee | 12 | 0.67128 | 0.63974 | 0.65513 |
| RandomCommittee | 13 | 0.66842 | 0.63193 | 0.64966 |
| RandomCommittee | 14 | 0.66871 | 0.63275 | 0.65023 |
| RandomCommittee | 15 | 0.66871 | 0.63275 | 0.65023 |
| RandomCommittee | 16 | 0.66988 | 0.63275 | 0.65079 |
| RandomCommittee | 17 | 0.6693 | 0.6311 | 0.64964 |
| RandomCommittee | 18 | 0.67077 | 0.63027 | 0.64989 |
| RandomCommittee | 19 | 0.67106 | 0.6311 | 0.65047 |
| RandomCommittee | 20 | 0.6696 | 0.63079 | 0.64962 |
| RandomCommittee | 21 | 0.67018 | 0.63193 | 0.65049 |
